# Diversity, Abundance, and Some Characteristics of Bacteria Isolated from Earth Material Consumed by Wild Animals at Kudurs in the Sikhote-Alin Mountains, Russia

**DOI:** 10.1155/2020/8811047

**Published:** 2020-12-07

**Authors:** Elena Lebedeva, Alexander Panichev, Natalya Kharitonova, Aleksei Kholodov, Kirill Golokhvast

**Affiliations:** ^1^Laboratory of Geochemistry of Hypergene Processes, Far East Geological Institute FEB RAS, Vladivostok, Russia; ^2^Laboratory of Ecology and Animal Protection, Pacific Geographical Institute FEB RAS, Vladivostok, Russia; ^3^SEC Nanotechnology, Far Eastern Federal University, Vladivostok, Russia

## Abstract

In this work, geochemical and microbiological studies were performed at kudurs in the southeastern part of the Sikhote-Alin mountain range and in the Sikhote-Alin Nature Reserve located in Primorsky Krai, Russia. It was found that the earth material eaten by wild animals in both sites is represented by clay-zeolite tuffs of dacite-rhyolite composition. In the earth material, Na is predominant in bioavailable macronutrients and Zn, light lanthanides, and Y in trace elements. Microbiological studies of geophagic earths revealed a wide range of heterotrophic and autotrophic aerobes and anaerobes involved in the conversion of carbon, nitrogen, and sulfur. Iron- and manganese-oxidizing bacteria and silicate bacteria were identified as well. The isolated pure cultures of heterotrophic bacteria were represented mainly by Gram-positive spore-forming large rods of *Bacillus* sp. and Gram-negative heterotrophic aerobic and facultative anaerobic microorganisms *Burkholderia* sp. and *Microvirgula aerodenitrificans*, which oxidize iron and reduce sulfate. The ability of the bacteria *M. aerodenitrificans* to reduce sulfates is shown for the first time. According to the literature, the isolated microorganisms are able to actively extract rare earth elements from earth materials, transforming them from the bioinert state to a state accessible to herbivorous mammals.

## 1. Introduction

An instinctive form of earth material consumption (or geophagy) is common among herbivorous animals in many regions of the world. It is accompanied by kudurs—natural complexes with characteristic traces of animal activity that exist for many centuries. The term “kudur” is borrowed from the vocabulary of Turkic nomadic cattle herders [1]. From it, the term “kudurite” was derived to indicate any type of earth eaten by animals at kudurs.

This article deals with the microbiological study of clay-zeolite earths consumed by wild animals at three kudurs in the Sikhote-Alin Mountains. Two of them are located in the southeast, and the third one in the northern part of this mountainous territory.

Our interest in microbiological characteristics of the mineral substances consumed by animals at kudurs aims to understand the cause of the geophagy phenomenon, which is still largely unknown. Based on previous studies by various authors, only a few hypotheses regarding the causes of geophagy have been proposed so far. The most common of them are the need for sodium and mineral sorbents to normalize the electrolyte balance in the digestive tract of animals during the spring transition of animals to green food [2–4]; the need to replenish the body's lack of Fe; replenishment of symbiont microorganisms, parasite control, and regulation of pH in the digestive tract [5–7]; and removal of chemical wastes from the body using mineral sorbents [8, 9]. More recently, based on the analysis of extensive data on the geochemistry of geophagic earths in various regions of the world, we proposed the rare-earth or “REE hypothesis” [10]. Its main point is that some elements from the light lanthanides group associated in nerve tissues and internal secretion gland enzymes can be replaced easily by heavy analogues, which, unlike the light ones, are unable to perform the functions necessary for the body. As a result, vital systems of the body can be affected, which manifests in decreasing adaptive capacity of the body to counteract adverse external factors (geochemical, cosmophysical, climatic, social, and others). In response to this, stress arises in the body, which forces animals to look for natural mineral regulatory substances that can be either a source of missing light REE or effective sorbents of their heavy analogues. The desire of herbivores to consume earthy substances enriched with Na, when applied to kudurs enriched in REE, can be explained by the fact that Na and REE are often paragenetically related in the composition of geophagic earths.

Since it is known that various groups of microorganisms participate in the digestion of food in animals, it is appropriate to assume that earth materials consumed by animals can be the source of some beneficial microorganisms. It is also possible that soil microorganisms can participate in the conversion of mineral forms of rare-earth elements into bio-mineral forms, after which they can be assimilated in the body of mammals. That is why we made an attempt to study the microbiological characteristics of the most typical kudurites in the Sikhote-Alin.

The article is focused on studying the abundance of main physiological groups of bacteria, on isolating the prevailing heterotrophic bacteria, and on studying their diversity and some morphological, physiological, and biochemical characteristics.

## 2. Materials and Methods

**Figure 1:**
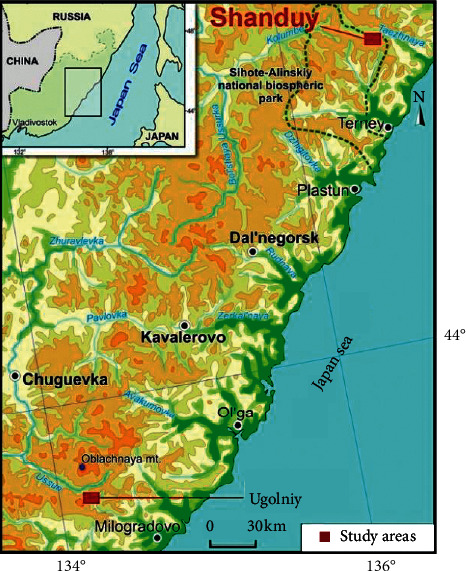
Location of sampling areas at kudurs in the Sikhote-Alin.

For microbiological studies, in 2017, we selected the samples of earths eaten by animals, which were collected at three kudurs. Two of them located on the Ugolniy stream at the head of the Milogradovka River, within the Zov Tigra (Call of the Tiger) National Park. The third kudur is located near the Shanduy Lakes in the territory of the Sikhote-Alin Nature Reserve (Figure 1).

Three microbiological samples were collected on the Ugolniy stream, including samples U-1 and U-2 from areas where animals eat earths at the Ugolniy-1 kudur. This kudur is a 10 × 15 m bedrock exposed by a landslide on a steep side of the stream. The outcrops are represented by a unit of clayed layered tuffaceous sedimentary rocks (tuff sandstones of dacite-rhyolite composition) with 1 m-thick interlayer of lignite. In the outcrop, there are several clearly visible depressions up to a meter deep in spots of weak groundwater outflow with characteristic traces of animals in the form of bites and licks.

The sample U-3 was collected from the area where animals eat earths at the Ugolniy-2 kudur, located 500 m downstream on the right bank. It is a gently sloping erosion funnel on the bank of the stream, about 15 m in diameter, with mud surface amid rock debris (tuff sandstones of rhyolite composition). The main animal bites and licks are located under the roots of trees in the upper part of the erosion funnel. The dimensions of the eaten earths are up to half a meter deep and a meter across.

Judging by the animal tracks in the mud areas and approaching paths, both kudurs are visited mainly by red deer (*Cervus elaphus*), and in lesser numbers, by Siberian roe deer (*Capreolus pygargus*).

In the Shanduy Lakes area, one microbiological sample (Sh-1) was collected from the area of animal bites and licks at the Shanduy kudur, which is located about 1000 m east of the Bolshoye Lake (Figure 1). This outcrop of loose rocks (landwaste and various-sized fragments of rhyolite tuff sandstones) displaced by a landslide is stretched along a gentle slope with an open area of 30 by 50 m. The areas where animals eat earths are located along the edges of a shallow erosive furrow crossing the site from top to bottom. Some deepenings in the eaten earths go half a meter deep. Judging by the animal tracks, the kudur is visited by red deer (*Cervus elaphus*) and elk (*Alces alces*).

The samples of kudurites were collected in sterile disposable containers. Prior to the laboratory analysis, the samples were stored in a refrigerator at 4°C for no more than 24 hours. The traditional methods of practical microbiology were used to identify and cultivate bacteria. The abundance of various physiological groups of bacteria was determined by the limiting dilution method in special selective media [11]. After incubation, based on the number of tubes in which growth was observed or not, the most probable number of cells contained in 1 gram of the initial substrate was calculated using McCready tables [12]. Pure cultures of saprophytic bacteria were isolated using Koch's plating method in meat infusion agar. Microorganisms were grown in an incubator at 25°C. Anaerobic forms of bacteria were cultivated in an anaerobic jar using the BD GasPak EZ anaerobe container system sachets. To study the morphology, sizes, motility, and spore formation of isolated pure bacterial cultures, a Carl Zeiss Axiostar Plus transmitted light microscope (Carl Zeiss, Germany) with AxioVision digital image processing software and phase contrast attachment (x1000) was used. Cell motility was tested by the hanging drop method. The type of the bacterial cell wall was determined using the Gram staining method [12]. To study the production of catalase, a drop of 3% hydrogen peroxide solution was added to a slide with the culture, with the positive reaction being the formation of gas bubbles. The oxidase enzyme was detected by the Ehrlich method in 24-hour cultures grown in meat infusion agar. Lecithinase activity was studied by plating the culture in salt egg yolk agar. Lecithinase activity was indicated by a golden yellow rim around the colony. Hydrolysis of starch was detected after treating the agar plate with Lugol's solution. The ability of the isolated heterotrophic bacteria to utilize monosaccharides and alcohols was studied in Hiss's media (Biocompas-C, Uglich, Russia) containing mannitol and glucose. Optimum growth temperatures of the isolated strains of bacteria *Bacillus* sp. and *Burkholderia* sp. were detected in meat infusion agar by streak culture in a Petri dish followed by incubation at 20°C, 35°C, 42°C, 45°C, and 50°C, taking note of the occurrence and intensity of bacterial growth. Enrichment cultures of sulfate-reducing bacteria (SRB) were obtained by plating the samples in Postgate's medium C with sodium lactate at the incubation temperature of 25°C. Pure culture of SRB was obtained by purification in media recommended by Postgate [11]. Some physiological and biochemical characteristics of SRB were determined by the ability of bacteria to use different sources of carbon: alcohols and organic acids. Organic acids and alcohols were added in concentrations of 3.5 g/L to Postgate's medium C without yeast extract. Sulfate, thiosulfate, and elemental sulfur at a concentration of 4.5 g/L were added to the medium as electron acceptors. The spore formation ability was tested by heating cell suspensions in a water bath at 80°C for 10, 20, and 30 minutes [13]. To evaluate the effect of temperature, salinity, and pH on the growth of SRB, Postgate's medium C was employed. The temperature at which the SRB strain is able to grow was tested using an incubator in the temperature range from 25°C to 55°C. The pH value was determined in Postgate's medium C without sodium chloride using a 10% hydrochloric acid solution or 10% sodium hydroxide solution. The effect of salinity on strain growth was determined by adding 1–6% of NaCl to the liquid Postgate's medium C. The results were recorded after 10–14 days of incubation at 25°C by H_2_S release. The microorganisms were identified to the genus according to Bergey's manual of determinative bacteriology [14], as well as using molecular genetic methods. Genomic DNA of the SRB strain was isolated from biomass obtained by centrifugation of 16 mL of pure culture at 5000 rpm for 20 minutes. Genomic DNA was isolated using the AxyPrep Bacterial Genomic DNA Miniprep Kit (Axygen, USA). PCR amplification of 16SrDNA was carried out using primers BF-20 (5′-AGAGTTTGATCA/CTGGCTCAG-3′) and BR2/22 (5′-TACGGTTACCTTGTTACGACTT-3′). Sequence homology search was performed on NCBI (http://blast.ncbi.nlm.nih.gov) and EMBL-EBI (http://www.ebi.ac.uk/ena) servers. Genome assembly, multiple sequence alignments, and genetic distance calculations were performed using MEGA software, version 6. Molecular genetic studies were carried out in the Pacific Institute of Bioorganic Chemistry FEB RAS.

To determine the yield of chemical elements from kudurites under acidic conditions in the abomasum of ruminant mammals, we treated the samples with hydrochloric acid extracts in the Geochemistry Laboratory of the Pacific Geographical Institute FEB RAS. The initial kudurite samples were air-dried, ground using a porcelain pestle, and sieved through a 1 mm sieve. The samples of dry geophagic earths weighing 5.00 g were sieved through a 1 mm sieve, and 50.00 mL of 0.1 N HCl was added to it, and then the mixture was shaken for 15 minutes and left for a day. The extracts were filtered using ash-free blue ribbon filters that were washed with hot 0.1 N HCl solution and the first portions of the sample. After that the extracts were transferred for the analysis of the content of macroelements and trace elements to the analytical laboratory of the Far East Geological Institute FEB RAS. The chemical composition of liquid samples was determined by mass spectrometry with inductively coupled plasma using an Agilent 7700x spectrometer (Agilent Technologies, Inc., USA). Some ions in liquid samples were determined by ion chromatography using an LC-20 liquid chromatograph (Shimadzu, Japan).

## 3. Results and Discussion

According to earlier data of mineralogical and geochemical studies of earths eaten by animals in both areas [15], these kudurites can be classified as clay-zeolite weathering products of dacite-rhyolite volcanic tuffs. The earths are composed of sand-size quartz crystals and feldspars (sodium feldspar, potash feldspar)—approximately 20 to 50% of the volume, clay minerals, predominantly smectite and alternating-layer illite-smectite (10 to 40% of the volume), and zeolites (heulandite and clinoptilolite). The zeolite content ranges from 20 to 40%.

Using acid extracts, it was found that Na prevails in bioavailable macroelements with a pure element yield of 3.2 to 5.7 grams per 1 kg of kudurite (Table 1).

**Figure 2:**
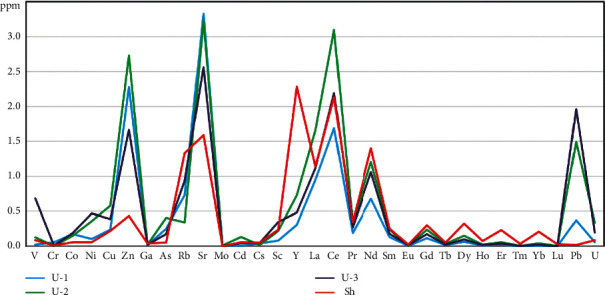
Trace elements extracted into HCl pH-1 acid extract, mg per 1 kg (ppm) of dry kudurite.

Among the trace elements, Zn, Sr, Pb, as well as Y, La, Ce, and Nd showed the highest acid extractability (Figure 2).

Microbiological studies revealed a large number of physiological groups of bacteria in the geophagic earths, on average ranging from 8.6 × 10^3^ (U-2) to 2.1 × 10^4^ (U-3) cells/g. In bacteria, saprophytes predominated significantly (Table 2), indicating the presence of organic matter in the earths. The number of aerobe saprophytes exceeded the number of anaerobes in all the analyzed samples (Table 2).

In the studied rocks, we also noted a rather high number of silicate bacteria (1.0 × 10^4^–2.2 × 10^4^ cells/g) and microorganisms of the nitrogen cycle, especially heterotrophic nitrate bacteria (1.2 × 10^3^–8.4 × 10^4^ cells/g), which indicates the ongoing decomposition of nitrogen-containing organic substances to ammonia and oxidation of nitrogen compounds, as well as the destruction of silicate minerals involving bacteria. A rather high number of manganese-oxidizing heterotrophic bacteria were found in the earth sample U-2 (1.5 × 10^4^ cells/g), which may be due to a higher content of manganese in these earths and more favorable conditions for the development of this group of bacteria. The number of anaerobic sulfate-reducing bacteria (SRB) in the earths varies (Table 2). The highest abundance of SRB was found in the sample Sh-1 (1.5 × 10^5^ cells/g), indicating ongoing sulfate reduction to hydrogen sulfide in the geophagic earth. In the studied earths, autotrophic nitrifying, iron-oxidizing, thionic, and heterotrophic manganese and iron-reducing bacteria were less abundant.

Heterotrophic bacteria isolated from all earth samples formed two types of colonies in selective media: milky gray colored, flat, shiny, 2–6 mm in diameter, and gray-beige colored, flat, dull, 5–10 mm in diameter. In the earth sample Sh-1, in addition to the main two types of bacterial colonies, we identified milk-beige colored colonies with a brown pigment, 2–4 mm in diameter. We isolated pure cultures (9 strains) and studied some of their morphological, physiological, and biochemical characteristics. The isolated strains of heterotrophic bacteria (all except strain 09.10) were Gram-positive, motile, spore-forming, catalase-positive, and rod-shaped with a cell size of 0.3–1.5/2–5 *µ*m (Table 3). The strains were able to oxidize glucose and mannitol, showed activity against starch hydrolysis, did not show lecithinase activity, and grew at 20–50°C (Table 3).

It is known that bacteria belonging to the genus *Bacillus* sp. are abundant in nature, inhabiting not only water, soils, and rocks but also intestines of humans and animals as a natural microflora. *Bacillus* spp. are capable of producing a wide range of biologically active substances; they have antagonistic activity against pathogenic and conditionally pathogenic bacteria, and they are also able to synthesize various lytic enzymes that break down polysaccharides, proteins, fats, and other macromolecules [16, 17]. Therefore, when consumed together with geophagic earths by wild animals, bacteria *Bacillus* sp. are likely to improve digestion of feed and contribute to the immunity of animals to infectious diseases, being a factor in the general protection of the body. There are also publications stating that various species of *Bacillus* sp. are capable of extracting REE from ores and minerals [18, 19, 52] and accumulating them in the cell wall during biosorption and bioaccumulation [20–28]. Bacteria belonging to the genus *Bacillus* are also well known for their ability to produce a wide range of siderophores [29, 30]. Some siderophores can chelate REE from aqueous solutions [31, 32] and igneous rocks [33, 34]. *Bacillus* spp. most likely can both enhance the efficiency of microbiological processes in the digestive tract of animals and participate in the accumulation of bioavailable REE forms in rocks, which may be one of the reasons for the instinctive eating of earth materials by wild animals.

However, it is also impossible to deny that the rocks being eaten can only be a source of minerals and trace elements for animals and the bacteria detected in the soil do not survive after entering into the animal's body. There is no clear answer to this question yet, and this will be shown by further research. A pure culture of active heterotrophic iron-oxidizing bacteria, which formed milky-beige colonies with brown pigment, was isolated from the Sh-1 earth material sample in the selective medium. The isolated bacteria were represented by Gram-negative, aerobic, motile, asporogenic, catalase-positive rods with a cell size of 0.5–1.0/3–5 *µ*m. Strain 09.10 grew at 20–35°C, and it was capable of starch hydrolysis and oxidation of glucose and mannitol (Table 3). Analysis of the nucleotide sequences of the 16S rRNA gene showed that strain 09.10 was 99.65% similar to bacteria *Burkholderia ambifaria*. It is known that many species of *Burkholderia* are typical inhabitants of soils and the root environment. A number of representatives are phytopathogenic organisms, as well as pathogenic agents in animals and humans (*B. cepacia*, *B. mallei*, and *B. pseudomallei*) [35]. It was reported that many species of *Burkholderia*, including *B. ambifaria*, have the potential for biodegradation of polyaromatic hydrocarbons and release biologically active volatile substances that have a stimulating effect on plants [36, 37]. There is evidence that bacteria of this genus were isolated from rocks, soils, and tailings characterized by increased REE concentrations [38, 39]. A study by Christophe et al. [40] reported that treatment of common pine roots with bacteria *Burkholderia* contributed to a significant release of macro- and micronutrients from apatites (including REE, especially Ce, La, and Nd). Therefore, *Burkholderia* spp., which we found in the earth material, can play an important role in the release of REE and other elements from rocks.

SRB were found in U-3 and Sh-1 earth samples; they were most abundant in the Sh-1 sample (Table 2). However, we could not isolate a pure SRB culture from the Sh-1 sample because the bacteria did not grow in the selective medium. We isolated a pure SRB culture from the U-3 sample and studied some of its morphological, physiological, and biochemical characteristics (Table 4).

The SRB sch strain was represented by Gram-negative, catalase- and oxidase-positive, non-spore-forming, thin, curved, very motile rods 0.3–0.5/1.1–2.3 *µ*m in size. In agar Postgate's medium C, the strain formed small, round black colonies, 0.5–1 mm in diameter. The isolate grew at of 25–40°C, pH = 5.0–8.0. It could grow in the presence of 1–5% NaCl (Table 3). The strain was identified using molecular genetic methods. According to the 16S rRNA gene sequencing, the nucleotide sequence of the SRB sch strain has 100% homology with the nucleotide sequence of *Microvirgula aerodenitrificans* DSM 15089 deposited in GenBank. The bacteria of the genus *Microvirgula* belong to the phylum Proteobacteria, domain Bacteria, and family Neisseriaceae.

This microorganism was first isolated from activated sludge and described by Patureau et al. in 1998 [41]. To date, only two species of this bacterium are known *M. aerodenitrificans* and *M. curvata*; the latter were isolated from soils contaminated with hydrocarbons [42]. Publications devoted to the study of bacteria *M. aerodenitrificans* are very few [41, 43]. From the available data, it is clear that *M. aerodenitrificans* were previously rarely isolated and are known as bacteria capable of denitrification. The sulfate reduction ability of bacteria *M. aerodenitrificans* was not noted in the literature before. The isolated strain of SRB sch *M. aerodenitrificans* grew in the Postgate-C medium at an optimal temperature of 25°C, pH = 7, and 0% NaCl concentration. Under these conditions, it was able to reduce sulfates to H_2_S for 10–14 days. The isolate of SRB sch *M. aerodenitrificans* is capable of using sulfate, thiosulfate, and nitrate as electron acceptors, and lactate, ethanol, acetate, and formate as electron donors (Table 4). Thus, the participation of the isolated bacteria *M. aerodenitrificans* in sulfate reduction processes was first revealed. According to the literature, SRB are common in nature, inhabiting not only rocks and earths but are also found in human feces and insect intestines, and isolated from animal rumen [44–49]. There are also studies showing that SRB can effectively mobilize REE from phosphate minerals [50, 51]. Thus, the isolated SRB *M. aerodenitrificans* contribute to the extraction of REE from earth materials, transforming it into a state accessible to animals.

Different physiological groups of heterotrophic aerobic and anaerobic microorganisms, relatively abundant in the studied earth material, produce various enzymes, organic acids, and siderophores, which can significantly affect the mobility of trace elements in soils (including REE), contributing to the accumulation of such forms thereof that are accessible to mammals. The data obtained indicate that the microbiological factor is one of the reasons for the consumption of earth materials by wild animals.

## 4. Conclusions

The study has shown that the clay-zeolite kudurites of the Sikhote-Alin are inhabited by various physiological groups of bacteria with average abundance. Aerobic saprophytic bacteria, microorganisms of the geochemical nitrogen cycle (especially heterotrophic nitrifiers and ammonifying bacteria), and silicate and sulfate-reducing bacteria are predominant there. The isolated pure cultures of heterotrophic bacteria were mainly represented by Gram-positive spore-forming large rods *Bacillus* sp. We also identified Gram-negative heterotrophic aerobic and facultative anaerobic microorganisms *Burkholderia* sp. and *M. aerodenitrificans*, which oxidize iron and reduce sulfates. In this work, we first showed the ability of the bacteria *M. aerodenitrificans* to reduce sulfates.

## Figures and Tables

**Table 1:** Concentrations of acid-soluble forms of elements in kudurite samples (ppm per air-dry sample).

Kudur	Sample	Ti	Al	Fe	Mn	Mg	Ca	Na	K	P
Ugolniy 1	U1	0.3	315	53	7	23	221	3217	194	1.6
	U2	3.3	140	127	79	101	1174	4633	152	2.0
Ugolniy 2	U3	0.1	198	104	125	171	1680	5724	288	51.0
Shanduy	Sh-1	0.1	4487	21	59	102	1220	5257	496	3.9

**Table 2:** Abundance of predominant physiological groups of bacteria in earth materials.

Physiological groups of bacteria, cells/g	Ugolniy-1		Ugolniy-2	Shanduy
	U-1	U-2	U-3	Sh-1
Saprophytic bacteria, aerobes	6.4 × 10^4^	5.2 × 10^4^	9.7 × 10^4^	8.7 × 10^4^
Saprophytic bacteria, anaerobes	1.3 × 10^4^	7.8 × 10^3^	7.7 × 10^4^	1.2 × 10^4^
Nitrogen-fixing bacteria	4.1 × 10^3^	7.4 × 10^3^	3.2 × 10^4^	0
Ammonifying bacteria	3.0 × 10^4^	1.5 × 10^4^	8.1 × 10^3^	1.1 × 10^4^
Heterotrophic nitrifiers	5.9 × 10^4^	3.2 × 10^4^	8.4 × 10^4^	1.2 × 10^3^
Denitrifiers	4.5 × 10^3^	2.5 × 10^2^	4.5 × 10^2^	5.5 × 10^2^
Sulfur-reducing bacteria	0	0	2.5 × 10^2^	1.5 × 10^5^
Iron-oxidizing bacteria, heterotrophs	2.7 × 10^2^	0	1.4 × 10^2^	6.1 × 10^3^
Manganese-oxidizing bacteria, heterotrophs	0.4 × 10^2^	1.5 × 10^4^	6.1 × 10^2^	4.1 × 10^3^
Silicate bacteria	2.2 × 10^4^	1.2 × 10^4^	1.1 × 10^4^	1.0 × 10^4^

**Table 3:** Comparative characteristics of some morphological, physiological, and biochemical characteristics of heterotrophic bacteria strains isolated from earth material samples.

Strain name	Isolated from	Cell morphology	Growth conditions	Motility	Spore formation	Catalase	Starch hydrolysis	Lecithinase	Mannitol	Glucose	T, °C min/max	Bacteria genus
15.09	U-1	Gram-positive rods 0.4–0.7/2–5 *µ*m	A, FAN	+	+	+	+	−	+	+	20/35	*Bacillus sp.*
26.09	U-1	Gram-positive rods 0.6–0.8/2–3 *µ*m	A, FAN	+	+	+	+	−	+	+	20/45	*Bacillus sp.*
07.10	U-2	Gram-positive rods 0.7–1.2/2–4 *µ*m	A, FAN	+	+	+	+	−	+	+	20/50	*Bacillus sp.*
14.10	U-2	Gram-positive rods 0.9–1.2/3–5 *µ*m	A, FAN	+	+	+	+	−	+	+	20/42	*Bacillus sp.*
13.10	U-3	Gram-positive rods 0.8–1.5/2–5 *µ*m	A, FAN	+	+	+	+	−	+	+	20/42	*Bacillus sp.*
02.11	U-3	Gram-positive rods 0.4–0.6/3–5 *µ*m	A, FAN	+	+	+	+	−	+	+	20/45	*Bacillus sp.*
09.10	Sh-1	Gram-negative rods 0.5–1.0/3–5 *µ*m	A	+	−	+	+	−	+	+	20/35	*Burkholderia sp.*
03.12	Sh-1	Gram-positive rods 0.7–1.0/2–5 *µ*m	A, FAN	+	+	+	+	−	+	+	20/35	*Bacillus sp.*
11.12	Sh-1	Gram-positive rods 0.3–0.6/2–4 *µ*m	A, FAN	+	+	+	+	−	+	+	20/42	*Bacillus sp.*

A, aerobes; FAN, facultative anaerobes.

**Table 4:** Some morphological, physiological, and biochemical characteristics of strain SRB sch isolated from U-3 earth material samples.

Traits		Characteristics	SRB sch
Cell morphology		Gram stain	*−*
		Cell shaper	Rods
		Cell size, *µ*m	0.3–0.5/1.1–2.3
		Motility	+
		Spore formation	*−*
Enzymes		Catalase	+
		Oxidase	+
Oxygen relationship		Facultative anaerobes	
Electron acceptor		Sulfate	+
		Thiosulfate	+
		Elemental sulfur	*−*
		Nitrate	+
Electron donor		Lactate	+
		Ethanol	+
		Acetate	+
		Citrate	*−*
		Formate	+
		Malonate	*−*
Growth at various temperatures, pH, NaCl concentrations	T, °C	25°C	+
		35°C	+
		40°C	+
		55°C	*−*
	pH	5.0	+
		6.0	+
		7.0	+
		8.0	+
		9.0	*−*
	NaCl	1%	+
		3%	+
		4%	+
		5%	+
		6%	*−*

+ Bacterial growth; *−* no bacterial growth.

## Data Availability

The data used to support the findings of this study are included within the article.
